# An exploration of O—H⋯O and C—H⋯π inter­actions in a long-chain-ester-substituted phenyl­phenol: methyl 10-[4-(4-hydroxyphenyl)phenoxy]decanoate

**DOI:** 10.1107/S2056989017016589

**Published:** 2018-04-17

**Authors:** David K. Geiger, H. Cristina Geiger, Dominic L. Morell

**Affiliations:** a Department of Chemistry, SUNY-College at Geneseo, Geneseo, NY 14454, USA

**Keywords:** crystal structure, Hirshfeld surface, energy framework, inter­action energy, hydrogen bonds, C—H⋯π inter­actions

## Abstract

An understanding of the driving forces resulting in crystallization *vs* organogel formation is essential to the development of modern soft materials. In the mol­ecular structure of the title compound, methyl 10-[4-(4-hydroxyphenyl)phen­oxy]decanoate (**MBO10Me**), C_23_H_30_O_4_, the aromatic rings of the biphenyl group are canted by 6.6 (2)° and the long-chain ester group has an extended conformation. In the crystal, mol­ecules are linked by O—H⋯O hydrogen bonds, forming chains along [10]. The chains are linked by C—H⋯O hydrogen bonds, forming layers parallel to the *ac* plane. The layers are linked by C—H⋯π inter­actions, forming a three-dimensional supra­molecular structure. The extended structure exhibits a lamellar sheet arrangement of mol­ecules stacking along the *b*-axis direction. Each mol­ecule has six nearest neighbors and the seven-mol­ecule bundles stack to form a columnar superstructure. Inter­action energies within the bundles are dominated by dispersion forces, whereas inter­columnar inter­actions have a greater electrostatic component.

## Abstract 2

The superstructure of 4-(9-methyl­oxycarbonyl­non­yloxy)phenyl­phenol is dominated by O—H⋯O and C—H⋯O hydrogen-bonding and C—H⋯π inter­actions. Hirshfeld surface, fingerprint plot, inter­action energy and energy framework analyses were used to explore the nature and strength of the inter­molecular inter­actions.

## Chemical context   

In a gel, the scaffold mol­ecules (the gelator) assemble into a network of fibers, which trap large numbers of solvent mol­ecules by way of non-covalent inter­actions (Weiss, 2014 ▸). Organogels, which are obtained by dissolving a small amount of a low-mol­ecular-mass organic gelator in an organic solvent, have myriad uses, including drug delivery and biomedical diagnostics (Wu & Wang, 2016 ▸; Tibbitt *et al.*, 2016 ▸), medical implants (Liow *et al.*, 2016 ▸; Yasmeen *et al.*, 2014 ▸), and tissue engineering (Xavier *et al.*, 2015 ▸; Yan *et al.*, 2015 ▸).

For a gel, self-assembly of a three-dimensional arrangement of mol­ecules incorporating a large number of solvent mol­ecules results in a thermodynamically stable state, whereas self-assembly followed by crystallization gives a solid. The factors resulting in gelation rather than crystallization are subtle and, as a result, there are few examples of single-crystal structure determinations of organogelators (Adhikari *et al.*, 2016 ▸; Rojek *et al.*, 2015 ▸; Cui *et al.*, 2010 ▸; Martin *et al.*, 2016 ▸; Geiger, Zick *et al.*, 2017 ▸; Geiger, Geiger *et al.*, 2017 ▸).

Traditional hydrogen bonding, van der Waals forces, and π–π and C—H⋯π inter­actions play important roles in determining the stability of organogels and crystalline lattices. The combination of solid-state structural data obtained *via* X-ray diffraction analysis and inter­action energies determined using computational techniques affords a powerful means of exploring the subtle differences in the driving force for crystallization *vs* gelation.
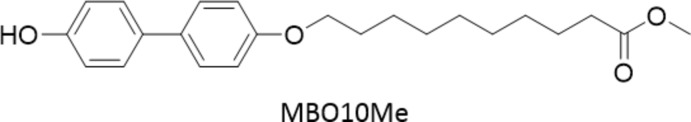


Recently, we reported the crystal structures and gelation properties of two bis­(long-chain-ester)-substituted biphenyl compounds (Geiger, Geiger *et al.*, 2017 ▸). To further understand the factors favoring gelation over crystallization, we have extended our exploration to a mono-substituted analog. In this report, we explore the structure, gelation ability, and inter­molecular inter­actions exhibited by methyl 10-[4-(4-hydroxyphenyl)phenoxy]decanoate (**MBO10Me**). Using *CrystalExplorer17* (Turner *et al.*, 2017 ▸), we have estimated the strengths of the primary inter­molecular inter­actions found in the supra­molecular structure. As expected, the presence of the phenol functional group results in an extended O—H⋯O and C–H⋯O hydrogen-bonding network. In addition, van der Waals forces and C—H⋯π inter­actions are observed.

## Structural commentary   

**MBO10Me** was isolated as a side product during the synthesis of the corresponding bis­(ester-substituted)biphenyl, 4,4′-bis­(9-methyl­oxycarbonyl­non­yloxy)biphenyl, BBO10Me (see Scheme below).
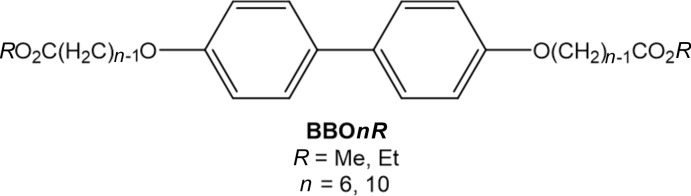


Although BBO10Me readily forms stable gels in a variety of solvents, **MBO10Me** does not behave as an organogelator in any of the solvents examined. The solid-state structures of BBO6Me and BBO6Et have been reported (Geiger, Geiger *et al.*, 2017 ▸). BBO6Me behaves as an organogelator, but BBO6Et does not. The two compounds are isostructural and a comparative energy framework analysis (Turner *et al.*, 2015 ▸) showed that the ethyl ester exhibits weaker inter­columnar inter­actions. The structural characterization of MBO10Me was undertaken in an effort to better understand the subtle differences in the strengths of the inter­molecular inter­actions that control gelation.

**Figure 1:**
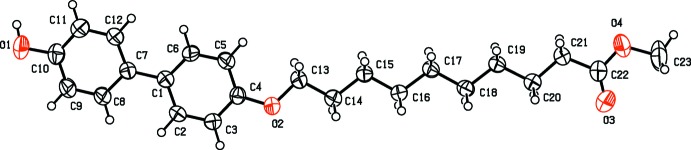
View of the mol­ecular structure of **MBO10Me**, showing the atom-labeling scheme. Displacement ellipsoids are drawn at the 50% probability level.

Fig. 1 ▸ shows the mol­ecular structure of **MBO10Me** with the atom-labeling scheme. The dihedral angle between the two phenyl rings is 6.6 (2) ° and the C6—C1—C7—C12 torsion angle is −6.3 (4)°. The ester chain adopts a straight-chain conformation, as is found in similar structures (Geiger, Zick *et al.*, 2017 ▸; Geiger, Geiger *et al.*, 2017 ▸), which maximizes the inter­molecular van der Waals inter­actions. The ester chain is, however, tilted out of the plane of the phenyl ring to which it is attached, with a C13—O2—C4—C3 torsion angle of 173.2 (3)°.

## Supra­molecular features   

**Figure 2:**
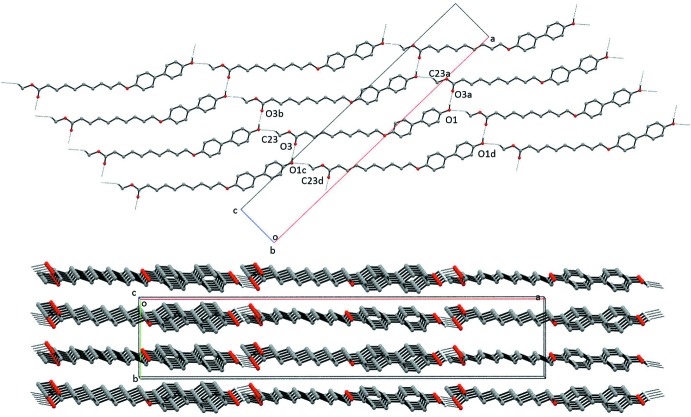
Two views of the packing in **MBO10Me** showing the layers parallel to (010). Only the H atoms involved in the O—H⋯O and C—H⋯O hydrogen bonds are shown. Symmetry codes: (*a*) *x* + 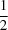, −*y* + 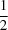, *z* − 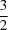; (*b*) *x*, *y z* + 1; (*c*) *x* − 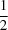, −*y* + 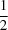, *z* + 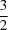; (*d*) *x*, *y*, *z* − 1.

**Figure 3:**
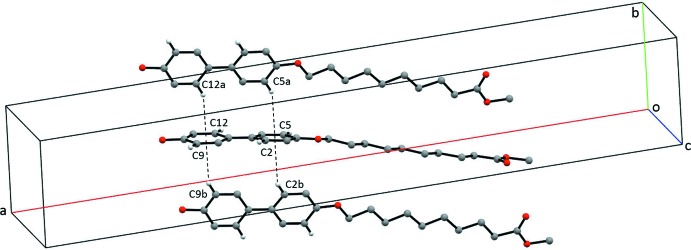
Partial crystal packing diagram of **MBO10Me**, emphasizing the C—H⋯π inter­actions. Only H atoms involved in these inter­actions are shown.

As seen in Table 1 ▸ and Fig. 2 ▸, O—H⋯O hydrogen bonds, in which the phenol group is the donor and the ester carbonyl group is the acceptor, and C—H⋯O hydrogen bonds, in which the methyl group is the donor and the phenol is the acceptor, result in sheets parallel to the *ac* plane that are composed of inter­linked 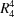
(52) rings. The structure is extended into the third dimension *via* C—H⋯π inter­actions involving phenyl ring hydrogen atoms and the π systems of both phenyl rings (see Fig. 3 ▸ and Table 1 ▸). The result is a columnar structure similar to that observed in BBO6Me and BBO6Et (Geiger, Geiger *et al.*, 2017 ▸) with an important difference: the columns are joined by an O—H⋯O hydrogen-bonding network in which the phenol is the donor and the ester carbonyl is the acceptor (Table 1 ▸ and Fig. 2 ▸).

## Database survey   

A search of the Cambridge Structural Database (CSD, V5.38, last update May 2017; Groom *et al.*, 2016 ▸) for 4,4′-biphenols yielded 21 structures, excluding those in which the biphenol was coordinated to a metal. There are 15 examples of structures with biphenol mol­ecules in which the dihedral angle between phenyl rings is 2° or less. [The calculated rotational barrier in the gas phase for 4,4′-biphenyl is *ca* 8 kJ mol^−1^ (Johansson & Olsen, 2008 ▸).] In the title compound, **MBO10Me**, the dihedral angle between the two phenyl rings is 6.6 (2)°.

## Hirshfeld surface analysis, inter­action energies   

**Figure 4:**
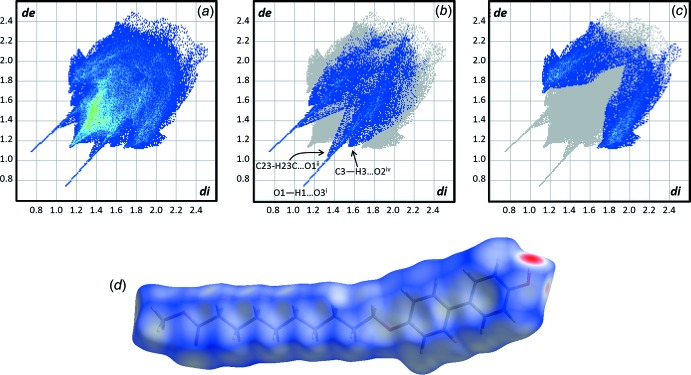
Fingerprint plots for **MBO10Me**, including (*a*) all inter­molecular contacts, (*b*) H⋯O inter­actions, (*c*) C—H⋯π inter­actions, and (*d*) Hirshfeld surface for **MBO10Me**.

Using *CrystalExplorer17* (Turner *et al.*, 2017 ▸), the Hirshfeld surface and fingerprint plots were calculated (see Section 9 for details). As seen in Fig. 4 ▸, the closest inter­molecular contacts involve the phenol group. Each of the types of hydrogen-bonding inter­actions are clearly discernible in the fingerprint plot. The presence of C—H⋯π bonding is also apparent. The H⋯O and H⋯C surface-contact coverages are 17.6% and 22.9%, respectively. No significant π–π inter­actions are are observed [the closest ring centroid-to-ring centroid distance is 4.921 (2) Å].

**Figure 5:**
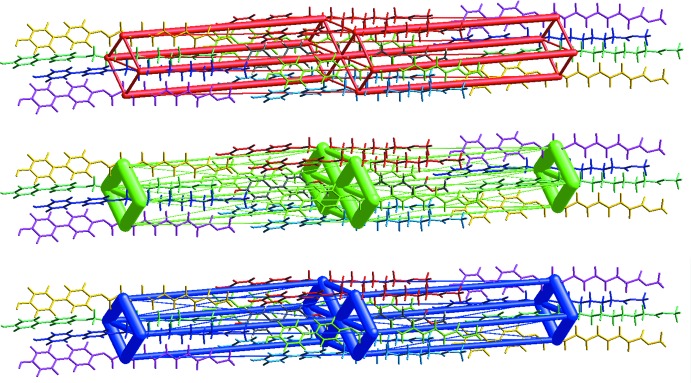
Energy framework diagram for separate electrostatic (top, red) and dispersion (middle, green) components of **MBO10Me** and the total inter­action energy (bottom, blue). The energy factor scale is 120 and the cut-off is 5.00 kJ mol^−1^.

Table 2 ▸ shows the results of the inter­action energy calculations (see *Section 9* for details). The results are represented graphically in Fig. 5 ▸ as framework energy diagrams (Turner *et al.*, 2015 ▸). In an energy framework, the cylinder size correlates to the strength of the inter­action. The framework is reminiscent of that observed in the bis­(substituted) compounds with inter­actions that are primarily dispersive in nature between the six nearest intra­columnar neighbors. However, the inter­columnar inter­actions, which possess the O—H⋯O hydrogen bonding, have greater electrostatic components. These findings show that the van der Waals and C—H⋯π inter­actions result in significantly favorable inter­molecular attractive forces, surpassing the strength of the inter­columnar O—H⋯O inter­action.

Based on the three structures reported to date, a columnar supra­molecular structure appears to be a common feature of long-chain ester compounds with a biphenyl core. The findings reported herein support the rationale posited for the difference in gelation ability exhibited by BBO6Me and BBO6Et (Geiger, Geiger *et al.*, 2017 ▸), *i.e.*, the strength of the inter­columnar inter­actions. The O—H⋯O hydrogen bonds between columns in **MBO10Me** are about twice the strength of the inter­columnar inter­actions found in BBO6Me (−15.5 kJ mol^−1^) and three times that found in BBO6Et (−10.1 kJ mol^−1^). A possible explanation for the lack of gelation ability of **MBO10Me** is that the stronger inter­columnar inter­actions favor formation of the crystal lattice rather than incorporation of a large number of solvent mol­ecules giving a gel.

## Synthesis and crystallization   

### Methyl 10-[4-(4-hydroxy­phenyl)phen­oxy]decan­oate (MBO10Me)   

The title compound was isolated as a minor side-product during the synthesis of the organogelator 4,4′-bis-(9-methyl­oxycarbonyl­non­yloxy)biphenyl (BBO10Me). ^1^H NMR (400 MHz, DMSO-d_6_) δ 9.40 (*s*, 1H), 7.44 (*d*, 2H), 7.38 (*d*, 2H), 6.92 (*d*, 2H), 6.68 (*d*, 2H), 4.02 (*t*, 2H), 3.60 (*s*, 3H), 2.20 (*t*, 2H), 1.73 (*m*, 2H), 1.35–1.45 (*m*, 12H). Single crystals suitable for X-ray analysis were isolated from the NMR tube in DMSO-*d*_6_.

## Gelation studies   

The gelation behavior of **MBO10Me** was examined in *n*-octa­nol, *n*-hexa­nol, *n*-butanol and ethanol. Gelation attempts were carried out using a 2.0% (*wt*/*wt*) of the compound and solvent in a screw-capped vial. The mixture was heated until all the solid dissolved and was then allowed to cool to room temperature. Formation of a gel is indicated when inversion of the vial yields no movement of the solvent.

## Refinement   

Crystal data, data collection and structure refinement details are summarized in Table 3 ▸. All H atoms were located in difference-Fourier maps. H atoms were refined using a riding model, with C—H = 0.95 Å and *U*_iso_(H) = 1.2*U*_eq_(C) for the aromatic positions, C—H = 0.99 Å and *U*_iso_(H) = 1.2*U*_eq_(C) for the methyl­ene groups, and C—H = 0.98 Å and *U*_iso_(H) = 1.5*U*_eq_(C) for the methyl group. The phenolic H atom was refined freely, including the isotropic displacement parameter. A meaningless Flack parameter and corresponding standard deviation were observed.

## Hirshfeld surface, fingerprint plots, inter­action energy calculations   

Hirshfeld surfaces, fingerprint plots, inter­action energies and energy frameworks (Turner *et al.*, 2015 ▸) were calculated using *CrystalExplorer17* (Turner *et al.*, 2017 ▸). Inter­action energies were calculated employing the CE-B3LYP/6-31G(*d,p*) functional/basis set combination and are corrected for basis set superposition energy using the counterpoise method. The inter­action energy is broken down as

*E*_tot_ = *k*_ele_ *E′*_ele_ + *k*_pol_ *E′*_pol_ + *k*_dis_ *E′*_dis_ + *k*_rep_ *E′*_rep_where the *k* values are scale factors, *E′*_ele_ represents the electrostatic component, *E′*_pol_ the polarization energy, *E′*_dis_ the dispersion energy, and *E′*_rep_ the exchange-repulsion energy (Turner *et al.*, 2014 ▸; Mackenzie *et al.*, 2017 ▸). The C—H bond lengths were converted to normalized values based on neutron diffraction results (Allen *et al.*, 2004 ▸).

## Supplementary Material

Crystal structure: contains datablock(s) global, I. DOI: 10.1107/S2056989017016589/su5401sup1.cif

Structure factors: contains datablock(s) I. DOI: 10.1107/S2056989017016589/su5401Isup2.hkl

Supporting information file. DOI: 10.1107/S2056989017016589/su5401Isup3.mol

Supporting information file. DOI: 10.1107/S2056989017016589/su5401Isup4.cml

CCDC reference: 1586244

Additional supporting information:  crystallographic information; 3D view; checkCIF report

## Figures and Tables

**Table 1:** Hydrogen-bond geometry (Å, °) *Cg*1 and *Cg*2 are the centroids of rings C1–C6 and C7–C12, respectively.

*D*—H⋯*A*	*D*—H	H⋯*A*	*D*⋯*A*	*D*—H⋯*A*
O1—H1⋯O3^i^	0.89 (4)	1.96 (4)	2.813 (5)	162 (4)
C23—H23*C*⋯O1^ii^	0.98	2.46	3.149 (5)	127
C23—H23*A*⋯O3^iii^	0.98	2.74	3.564 (6)	142
C3—H3⋯O2^iv^	0.95	2.82	3.627 (4)	143
C2—H2⋯*Cg*1^iv^	0.95	2.98	3.737 (4)	138
C9—H9⋯*Cg*1^iv^	0.95	2.89	3.716 (4)	146
C5—H5⋯*Cg*2^v^	0.95	2.95	3.722 (4)	139
C12—H12⋯*Cg*2^v^	0.95	2.83	3.661 (4)	147

Symmetry codes: (i) 
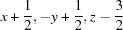
; (ii) 
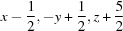
; (iii) 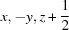; (iv) 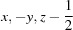; (v) 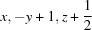.

**Table 2:** Inter­action energies *N* refers to the number of mol­ecules with an *R* mol­ecular centroid-to-centroid distance (Å). Energies are in kJ mol^−1^.

*N*	primary inter­action	*R*	*E*′_ele_	*E*′_pol_	*E*′_dis_	*E*′_rep_	*E*_tot_
2	C—H⋯π	4.91	−13.6	−2.8	−83.5	43.2	−62.5
2	C—H⋯π	4.98	−13.5	−3.5	−76.1	38.7	−59.2
2	H⋯H	6.70	−8.2	−1.2	−38.2	18.1	−31.7
2	O1—H⋯O3	23.60	−34.2	−7.1	−10.6	33.0	−30.3
2	C—H⋯O1	27.25	−6.1	−1.3	−5.5	8.8	−6.8
2	C—H⋯O1	25.53	−1.9	−0.4	−4.4	1.6	−5.1

Scale factors used to determine *E*_tot_: *k*_ele_ = 1.057, *k*_pol_ = 0.740, *k*_dis_ = 0.871, *k*_rep_ = 0.618 (Mackenzie *et al.*, 2017 ▸). See Section 9 for calculation details.

**Table 3:** Experimental details

Crystal data	
Chemical formula	C_23_H_30_O_4_
*M*_r_	370.47
Crystal system, space group	Monoclinic, *C* *c*
Temperature (K)	200
*a*, *b*, *c* (Å)	42.287 (9), 7.2848 (15), 6.7006 (13)
β (°)	91.226 (12)
*V* (Å^3^)	2063.7 (7)
*Z*	4
Radiation type	Mo *K*α
μ (mm^−1^)	0.08
Crystal size (mm)	0.40 × 0.40 × 0.20

Data collection	
Diffractometer	Bruker SMART X2S benchtop
Absorption correction	Multi-scan (*SADABS*; Bruker, 2013 ▸)
*T*_min_, *T*_max_	0.64, 0.98
No. of measured, independent and observed [*I* > 2σ(*I*)] reflections	10387, 3095, 2363
*R*_int_	0.060
(sin θ/λ)_max_ (Å^−1^)	0.602

Refinement	
*R*[*F* ^2^ > 2σ(*F* ^2^)], *wR*(*F* ^2^), *S*	0.044, 0.122, 1.05
No. of reflections	3095
No. of parameters	249
No. of restraints	2
H-atom treatment	H atoms treated by a mixture of independent and constrained refinement
Δρ_max_, Δρ_min_ (e Å^−3^)	0.12, −0.18

Computer programs: *APEX2* and *SAINT* (Bruker, 2013 ▸), *SHELXS97* (Sheldrick, 2008 ▸), *SHELXL2014/7* (Sheldrick, 2015 ▸), *PLATON* (Spek, 2009 ▸), *Mercury* (Macrae *et al.*, 2008 ▸) and *publCIF* (Westrip, 2010 ▸).

## supplementary crystallographic information

### Crystal data

**Table:**

C_23_H_30_O_4_	*F*(000) = 800
*M**_r_* = 370.47	*D*_x_ = 1.192 Mg m^−^^3^
Monoclinic, *C**c*	Mo *K*α radiation, λ = 0.71073 Å
*a* = 42.287 (9) Å	Cell parameters from 122 reflections
*b* = 7.2848 (15) Å	θ = 3.1–19.4°
*c* = 6.7006 (13) Å	µ = 0.08 mm^−^^1^
β = 91.226 (12)°	*T* = 200 K
*V* = 2063.7 (7) Å^3^	Plate, clear colorless
*Z* = 4	0.40 × 0.40 × 0.20 mm

### Data collection

**Table:**

Bruker SMART X2S benchtop diffractometer	3095 independent reflections
Radiation source: sealed microfocus tube	2363 reflections with *I* > 2σ(*I*)
Doubly curved silicon crystal monochromator	*R*_int_ = 0.060
Detector resolution: 8.3330 pixels mm^-1^	θ_max_ = 25.3°, θ_min_ = 2.8°
ω scans	*h* = −44→50
Absorption correction: multi-scan (SADABS; Bruker, 2013)	*k* = −8→8
*T*_min_ = 0.64, *T*_max_ = 0.98	*l* = −8→8
10387 measured reflections	

### Refinement

**Table:**

Refinement on *F*^2^	Primary atom site location: structure-invariant direct methods
Least-squares matrix: full	Secondary atom site location: difference Fourier map
*R*[*F*^2^ > 2σ(*F*^2^)] = 0.044	Hydrogen site location: mixed
*wR*(*F*^2^) = 0.122	H atoms treated by a mixture of independent and constrained refinement
*S* = 1.05	*w* = 1/[σ^2^(*F*_o_^2^) + (0.063*P*)^2^ + 0.2432*P*] where *P* = (*F*_o_^2^ + 2*F*_c_^2^)/3
3095 reflections	(Δ/σ)_max_ < 0.001
249 parameters	Δρ_max_ = 0.12 e Å^−^^3^
2 restraints	Δρ_min_ = −0.18 e Å^−^^3^

### Special details

**Table:**

Geometry. All esds (except the esd in the dihedral angle between two l.s. planes) are estimated using the full covariance matrix. The cell esds are taken into account individually in the estimation of esds in distances, angles and torsion angles; correlations between esds in cell parameters are only used when they are defined by crystal symmetry. An approximate (isotropic) treatment of cell esds is used for estimating esds involving l.s. planes.

### Fractional atomic coordinates and isotropic or equivalent isotropic displacement parameters (Å^2^)

**Table:**

	*x*	*y*	*z*	*U*_iso_*/*U*_eq_	
O1	0.73167 (7)	0.2440 (4)	−0.5378 (5)	0.0736 (9)	
H1	0.7463 (10)	0.294 (5)	−0.458 (7)	0.077 (14)*	
O2	0.51725 (5)	0.2275 (3)	0.0196 (3)	0.0512 (7)	
O3	0.28496 (7)	0.1690 (4)	1.2022 (5)	0.0826 (9)	
O4	0.29945 (7)	0.3224 (4)	1.4752 (4)	0.0800 (9)	
C1	0.60847 (8)	0.2531 (4)	−0.2156 (5)	0.0383 (8)	
C2	0.58278 (8)	0.1710 (5)	−0.3145 (5)	0.0442 (8)	
H2	0.5858	0.1167	−0.4415	0.053*	
C3	0.55318 (8)	0.1662 (5)	−0.2339 (5)	0.0447 (8)	
H3	0.5364	0.108	−0.3058	0.054*	
C4	0.54735 (8)	0.2444 (5)	−0.0505 (5)	0.0401 (9)	
C5	0.57240 (9)	0.3313 (5)	0.0505 (5)	0.0508 (9)	
H5	0.569	0.3892	0.1753	0.061*	
C6	0.60217 (8)	0.3326 (4)	−0.0319 (6)	0.0499 (9)	
H6	0.619	0.3903	0.0402	0.06*	
C7	0.64072 (7)	0.2542 (4)	−0.3000 (5)	0.0392 (8)	
C8	0.64804 (9)	0.1590 (5)	−0.4734 (6)	0.0600 (10)	
H8	0.6317	0.0942	−0.5427	0.072*	
C9	0.67810 (9)	0.1556 (5)	−0.5479 (6)	0.0662 (11)	
H9	0.6822	0.0868	−0.665	0.079*	
C10	0.70209 (8)	0.2498 (4)	−0.4554 (6)	0.0522 (10)	
C11	0.69596 (9)	0.3481 (5)	−0.2861 (6)	0.0600 (10)	
H11	0.7124	0.4153	−0.2207	0.072*	
C12	0.66573 (8)	0.3490 (5)	−0.2107 (5)	0.0552 (10)	
H12	0.6619	0.4174	−0.0929	0.066*	
C13	0.51112 (8)	0.2887 (5)	0.2186 (5)	0.0469 (9)	
H13A	0.5118	0.4244	0.2255	0.056*	
H13B	0.5272	0.2385	0.3135	0.056*	
C14	0.47868 (8)	0.2203 (5)	0.2700 (6)	0.0483 (9)	
H14A	0.4788	0.0844	0.2685	0.058*	
H14B	0.4633	0.2624	0.1663	0.058*	
C15	0.46794 (8)	0.2860 (4)	0.4725 (5)	0.0437 (8)	
H15A	0.4682	0.4219	0.4748	0.052*	
H15B	0.4831	0.2421	0.5766	0.052*	
C16	0.43495 (8)	0.2191 (4)	0.5219 (5)	0.0426 (8)	
H16A	0.4199	0.2639	0.4178	0.051*	
H16B	0.4348	0.0832	0.5171	0.051*	
C17	0.42356 (8)	0.2799 (4)	0.7235 (5)	0.0429 (8)	
H17A	0.424	0.4157	0.7289	0.052*	
H17B	0.4385	0.2338	0.8276	0.052*	
C18	0.39041 (9)	0.2150 (4)	0.7728 (5)	0.0448 (8)	
H18A	0.3754	0.261	0.6691	0.054*	
H18B	0.3899	0.0792	0.7683	0.054*	
C19	0.37950 (8)	0.2779 (4)	0.9753 (5)	0.0442 (8)	
H19A	0.3811	0.4134	0.9811	0.053*	
H19B	0.3942	0.2279	1.0785	0.053*	
C20	0.34601 (8)	0.2228 (5)	1.0281 (5)	0.0469 (9)	
H20A	0.331	0.2703	0.9249	0.056*	
H20B	0.3444	0.0873	1.0291	0.056*	
C21	0.33705 (9)	0.2965 (5)	1.2292 (6)	0.0546 (10)	
H21A	0.3522	0.2475	1.3304	0.066*	
H21B	0.3396	0.4316	1.2272	0.066*	
C22	0.30427 (9)	0.2536 (5)	1.2951 (6)	0.0529 (9)	
C23	0.26842 (13)	0.2933 (7)	1.5605 (9)	0.0984 (18)	
H23A	0.2635	0.1618	1.5605	0.148*	
H23B	0.2524	0.3589	1.4805	0.148*	
H23C	0.2685	0.3397	1.6978	0.148*	

### Atomic displacement parameters (Å^2^)

**Table:**

	*U*^11^	*U*^22^	*U*^33^	*U*^12^	*U*^13^	*U*^23^
O1	0.0492 (17)	0.092 (2)	0.080 (2)	−0.0003 (13)	0.0247 (15)	−0.0105 (16)
O2	0.0425 (14)	0.0684 (17)	0.0429 (15)	−0.0038 (12)	0.0052 (12)	−0.0054 (12)
O3	0.0494 (15)	0.109 (2)	0.090 (2)	−0.0115 (16)	0.0097 (14)	−0.0197 (19)
O4	0.076 (2)	0.100 (2)	0.0656 (19)	−0.0162 (16)	0.0320 (16)	−0.0190 (17)
C1	0.046 (2)	0.0295 (16)	0.040 (2)	0.0002 (12)	0.0028 (15)	0.0012 (13)
C2	0.048 (2)	0.0502 (19)	0.0340 (19)	−0.0005 (15)	0.0007 (15)	−0.0056 (15)
C3	0.0430 (18)	0.049 (2)	0.042 (2)	−0.0037 (14)	−0.0013 (15)	−0.0019 (15)
C4	0.0399 (19)	0.0411 (19)	0.040 (2)	0.0010 (14)	0.0033 (16)	0.0048 (15)
C5	0.054 (2)	0.052 (2)	0.047 (2)	−0.0067 (16)	0.0087 (17)	−0.0128 (17)
C6	0.045 (2)	0.0500 (18)	0.055 (2)	−0.0121 (14)	0.0052 (15)	−0.0159 (16)
C7	0.045 (2)	0.0299 (16)	0.043 (2)	0.0006 (12)	0.0043 (16)	−0.0003 (13)
C8	0.055 (2)	0.066 (2)	0.060 (2)	−0.0132 (16)	0.0147 (18)	−0.0250 (19)
C9	0.061 (2)	0.071 (2)	0.066 (3)	−0.0080 (19)	0.021 (2)	−0.0298 (19)
C10	0.048 (2)	0.049 (2)	0.060 (3)	0.0047 (15)	0.0116 (18)	0.0014 (17)
C11	0.047 (2)	0.069 (2)	0.064 (3)	−0.0045 (16)	0.0020 (19)	−0.012 (2)
C12	0.048 (2)	0.067 (2)	0.051 (2)	−0.0021 (16)	0.0061 (18)	−0.0191 (17)
C13	0.047 (2)	0.053 (2)	0.040 (2)	−0.0002 (15)	0.0056 (16)	−0.0014 (16)
C14	0.048 (2)	0.051 (2)	0.046 (2)	−0.0051 (15)	0.0042 (16)	−0.0068 (17)
C15	0.0403 (18)	0.051 (2)	0.040 (2)	0.0011 (15)	0.0004 (15)	−0.0007 (16)
C16	0.0392 (18)	0.0465 (19)	0.042 (2)	−0.0024 (14)	0.0000 (15)	−0.0047 (16)
C17	0.0442 (19)	0.0486 (19)	0.0359 (19)	0.0004 (15)	−0.0003 (15)	0.0005 (16)
C18	0.0471 (19)	0.047 (2)	0.040 (2)	0.0001 (15)	−0.0005 (14)	−0.0055 (17)
C19	0.044 (2)	0.0509 (19)	0.0374 (19)	−0.0005 (15)	−0.0007 (16)	0.0000 (16)
C20	0.045 (2)	0.052 (2)	0.043 (2)	−0.0011 (15)	0.0009 (16)	−0.0029 (17)
C21	0.050 (2)	0.070 (3)	0.043 (2)	−0.0068 (17)	0.0075 (17)	−0.0075 (18)
C22	0.049 (2)	0.055 (2)	0.056 (3)	0.0026 (17)	0.0095 (18)	0.002 (2)
C23	0.090 (4)	0.100 (4)	0.108 (5)	−0.009 (3)	0.061 (3)	−0.008 (3)

### Geometric parameters (Å, º)

**Table:**

O1—C10	1.378 (4)	C13—H13A	0.99
O1—H1	0.89 (4)	C13—H13B	0.99
O2—C4	1.372 (4)	C14—C15	1.518 (5)
O2—C13	1.435 (4)	C14—H14A	0.99
O3—C22	1.188 (5)	C14—H14B	0.99
O4—C22	1.327 (5)	C15—C16	1.521 (4)
O4—C23	1.458 (5)	C15—H15A	0.99
C1—C6	1.391 (5)	C15—H15B	0.99
C1—C2	1.395 (4)	C16—C17	1.511 (4)
C1—C7	1.487 (3)	C16—H16A	0.99
C2—C3	1.374 (4)	C16—H16B	0.99
C2—H2	0.95	C17—C18	1.522 (4)
C3—C4	1.381 (5)	C17—H17A	0.99
C3—H3	0.95	C17—H17B	0.99
C4—C5	1.396 (5)	C18—C19	1.514 (5)
C5—C6	1.385 (5)	C18—H18A	0.99
C5—H5	0.95	C18—H18B	0.99
C6—H6	0.95	C19—C20	1.521 (4)
C7—C12	1.388 (4)	C19—H19A	0.99
C7—C8	1.394 (5)	C19—H19B	0.99
C8—C9	1.376 (5)	C20—C21	1.506 (5)
C8—H8	0.95	C20—H20A	0.99
C9—C10	1.363 (5)	C20—H20B	0.99
C9—H9	0.95	C21—C22	1.497 (5)
C10—C11	1.371 (5)	C21—H21A	0.99
C11—C12	1.385 (5)	C21—H21B	0.99
C11—H11	0.95	C23—H23A	0.98
C12—H12	0.95	C23—H23B	0.98
C13—C14	1.506 (4)	C23—H23C	0.98
			
C10—O1—H1	112 (3)	C14—C15—C16	112.8 (3)
C4—O2—C13	118.5 (2)	C14—C15—H15A	109.0
C22—O4—C23	117.3 (3)	C16—C15—H15A	109.0
C6—C1—C2	115.9 (3)	C14—C15—H15B	109.0
C6—C1—C7	121.9 (3)	C16—C15—H15B	109.0
C2—C1—C7	122.2 (3)	H15A—C15—H15B	107.8
C3—C2—C1	122.1 (3)	C17—C16—C15	114.3 (2)
C3—C2—H2	119.0	C17—C16—H16A	108.7
C1—C2—H2	119.0	C15—C16—H16A	108.7
C2—C3—C4	121.4 (3)	C17—C16—H16B	108.7
C2—C3—H3	119.3	C15—C16—H16B	108.7
C4—C3—H3	119.3	H16A—C16—H16B	107.6
O2—C4—C3	116.9 (3)	C16—C17—C18	114.6 (2)
O2—C4—C5	125.1 (3)	C16—C17—H17A	108.6
C3—C4—C5	118.0 (3)	C18—C17—H17A	108.6
C6—C5—C4	119.7 (3)	C16—C17—H17B	108.6
C6—C5—H5	120.1	C18—C17—H17B	108.6
C4—C5—H5	120.1	H17A—C17—H17B	107.6
C5—C6—C1	122.9 (3)	C19—C18—C17	113.6 (3)
C5—C6—H6	118.6	C19—C18—H18A	108.9
C1—C6—H6	118.6	C17—C18—H18A	108.9
C12—C7—C8	115.2 (3)	C19—C18—H18B	108.9
C12—C7—C1	122.3 (3)	C17—C18—H18B	108.9
C8—C7—C1	122.4 (3)	H18A—C18—H18B	107.7
C9—C8—C7	122.3 (3)	C18—C19—C20	115.6 (3)
C9—C8—H8	118.8	C18—C19—H19A	108.4
C7—C8—H8	118.8	C20—C19—H19A	108.4
C10—C9—C8	120.7 (3)	C18—C19—H19B	108.4
C10—C9—H9	119.6	C20—C19—H19B	108.4
C8—C9—H9	119.6	H19A—C19—H19B	107.4
C9—C10—C11	119.1 (3)	C21—C20—C19	111.5 (3)
C9—C10—O1	118.4 (3)	C21—C20—H20A	109.3
C11—C10—O1	122.5 (3)	C19—C20—H20A	109.3
C10—C11—C12	119.8 (3)	C21—C20—H20B	109.3
C10—C11—H11	120.1	C19—C20—H20B	109.3
C12—C11—H11	120.1	H20A—C20—H20B	108.0
C11—C12—C7	122.8 (3)	C22—C21—C20	116.2 (3)
C11—C12—H12	118.6	C22—C21—H21A	108.2
C7—C12—H12	118.6	C20—C21—H21A	108.2
O2—C13—C14	107.0 (3)	C22—C21—H21B	108.2
O2—C13—H13A	110.3	C20—C21—H21B	108.2
C14—C13—H13A	110.3	H21A—C21—H21B	107.4
O2—C13—H13B	110.3	O3—C22—O4	123.7 (4)
C14—C13—H13B	110.3	O3—C22—C21	125.7 (4)
H13A—C13—H13B	108.6	O4—C22—C21	110.5 (3)
C13—C14—C15	113.0 (3)	O4—C23—H23A	109.5
C13—C14—H14A	109.0	O4—C23—H23B	109.5
C15—C14—H14A	109.0	H23A—C23—H23B	109.5
C13—C14—H14B	109.0	O4—C23—H23C	109.5
C15—C14—H14B	109.0	H23A—C23—H23C	109.5
H14A—C14—H14B	107.8	H23B—C23—H23C	109.5
			
C6—C1—C2—C3	−1.0 (4)	C8—C9—C10—O1	−179.0 (3)
C7—C1—C2—C3	178.3 (3)	C9—C10—C11—C12	0.6 (5)
C1—C2—C3—C4	0.5 (5)	O1—C10—C11—C12	179.8 (3)
C13—O2—C4—C3	173.2 (3)	C10—C11—C12—C7	−0.3 (6)
C13—O2—C4—C5	−5.6 (5)	C8—C7—C12—C11	−0.8 (5)
C2—C3—C4—O2	−178.1 (3)	C1—C7—C12—C11	178.8 (3)
C2—C3—C4—C5	0.8 (5)	C4—O2—C13—C14	−169.0 (3)
O2—C4—C5—C6	177.1 (3)	O2—C13—C14—C15	−176.1 (3)
C3—C4—C5—C6	−1.7 (5)	C13—C14—C15—C16	179.1 (3)
C4—C5—C6—C1	1.2 (5)	C14—C15—C16—C17	179.3 (3)
C2—C1—C6—C5	0.2 (5)	C15—C16—C17—C18	179.4 (3)
C7—C1—C6—C5	−179.2 (3)	C16—C17—C18—C19	−179.9 (3)
C6—C1—C7—C12	−6.3 (4)	C17—C18—C19—C20	177.7 (2)
C2—C1—C7—C12	174.4 (3)	C18—C19—C20—C21	−178.3 (3)
C6—C1—C7—C8	173.2 (3)	C19—C20—C21—C22	178.9 (3)
C2—C1—C7—C8	−6.1 (4)	C23—O4—C22—O3	−0.9 (6)
C12—C7—C8—C9	1.6 (5)	C23—O4—C22—C21	179.6 (4)
C1—C7—C8—C9	−178.0 (3)	C20—C21—C22—O3	−0.8 (6)
C7—C8—C9—C10	−1.4 (6)	C20—C21—C22—O4	178.8 (3)
C8—C9—C10—C11	0.2 (6)		

### Hydrogen-bond geometry (Å, º)

Cg1 and Cg2 are the centroids of rings C1–C6 and C7–C12, respectively.

**Table:**

*D*—H···*A*	*D*—H	H···*A*	*D*···*A*	*D*—H···*A*
O1—H1···O3^i^	0.89 (4)	1.96 (4)	2.813 (5)	162 (4)
C23—H23*C*···O1^ii^	0.98	2.46	3.149 (5)	127
C23—H23*A*···O3^iii^	0.98	2.74	3.564 (6)	142
C3—H3···O2^iv^	0.95	2.82	3.627 (4)	143
C2—H2···*Cg*1^iv^	0.95	2.98	3.737 (4)	138
C9—H9···*Cg*1^iv^	0.95	2.89	3.716 (4)	146
C5—H5···*Cg*2^v^	0.95	2.95	3.722 (4)	139
C12—H12···*Cg*2^v^	0.95	2.83	3.661 (4)	147

Symmetry codes: (i) *x*+1/2, −*y*+1/2, *z*−3/2; (ii) *x*−1/2, −*y*+1/2, *z*+5/2; (iii) *x*, −*y*, *z*+1/2; (iv) *x*, −*y*, *z*−1/2; (v) *x*, −*y*+1, *z*+1/2.
